# Concordance of molecular microbiology and conventional culture techniques for infected diabetic foot ulcer management

**DOI:** 10.1111/dme.70089

**Published:** 2025-07-09

**Authors:** Angela Oates, Sarah T. Brown, Colin C. Everett, Fran Game, Jane E. Nixon, Tim Sloan, Michelle M. Lister, Michael Backhouse, Benjamin A. Lipsky, David Russell, Howard Collier, Joanna Dennett, Rachael Gilberts, E. Andrea Nelson, Jon Deeks, Sue Mallett, Roger Gasdby, Jane Lewis, Julie Brittenden, Andrew Bradbury, Jo Paton, Zoe Hoare, Deborah Fitzsimmons, Mark Wilcox, Gerry Rayman, Brenda Riley, Christine Thomas, Ravikanth Gouni, Haroon Siddique, Gillian A. Lomax, Joseph M. Pappachan, Satyan Rajbhandari, David Russell, Mushtaqur Rahman, Ryan D’Costa, Biswa Mishra, Zoe Boulton, Rajiv Gandhi, Anna Goodman, Vasileios Tsadlidis, Wendy Stoker, Kilimangalam Narayanan, Trudi Keast, Julie Wiper, Lynne Calland, Jennifer McCafferty, Scarlett Shaw, Angela Pearsall, Carol Gascoigne

**Affiliations:** ^1^ Leeds Beckett University Leeds UK; ^2^ CTRU Faculty of Medicine and Health University of Leeds Leeds UK; ^3^ University Hospitals of Derby and Burton NHS Foundation Trust Derby UK; ^4^ Health Sciences, Faculty of Medicine and Health University of Leeds Leeds UK; ^5^ University of Nottingham Nottingham UK; ^6^ Nottingham University Hospitals NHS Trust Nottingham UK; ^7^ Warwick Clinical Trials Unit University of Warwick Warwick UK; ^8^ University of Washington Seattle Washington State USA; ^9^ Faculty of Medicine and Health University of Leeds Leeds UK; ^10^ Glasgow Caledonian University Glasgow UK

**Keywords:** 16S ribosomal RNA, diabetic foot, microbiology, wound infection

## Abstract

**Aim:**

The management of infected diabetic foot ulcers (DFUs) requires balancing the need for timely interventions against the desire for targeted antibiotic therapy, which relies on laboratory results. This study aimed to evaluate concordance between molecular and conventional culture and sensitivity (C&S) methods in identifying bacteria from infected DFUs.

**Methods:**

This study was conducted alongside CODIFI2, a Phase III randomised controlled trial comparing tissue sampling with wound swabbing. It assessed C&S and metagenomic 16S rRNA gene sequencing (M16S) in DFUs with suspected mild or moderate infections using both tissue and swab samples.

**Results:**

In 145 participants, C&S identified 248 microorganisms across 25 genera including eight anaerobic genera. M16S identified a greater number and diversity of microorganisms, detecting 455 across 40 genera, including 173 anaerobes from 15 distinct genera. No bacterial growth was reported in 25.5% (95% CI: 18.0%–32.3%) of C&S samples, whereas M16S identified at least one organism in all samples. While the observed agreement between methods was high (75%), Cohen's Kappa revealed low concordance overall, except for *Pseudomonas* spp. and *Streptococcus* spp. (Kappa ≥ 0.5).

**Conclusions:**

M16S detected a broader microbial spectrum, including fastidious anaerobes, but its low concordance with C&S and lack of antibiotic sensitivity data, challenge its suitability as a replacement for C&S in mild or moderate DFU infections. It may offer advantages in infections where increased sensitivity is beneficial, particularly where extended culture approaches are recommended to detect fastidious or low‐abundance organisms. For mild to moderate DFU infections, our findings support continued reliance on conventional C&S testing.

**What's new?**

**What is already known about this subject?**

- Guidelines for managing diabetic foot ulcers (DFUs) recommend empirical antibiotics initially, with adjustments based on microbiology results for optimal antimicrobial stewardship.
- Conventional culture and sensitivity (C&S) methods take 48–72 h.
- Molecular microbiology can potentially provide faster results and detect a broader range of organisms.

**What this study found?**

- Molecular methodologies identified a greater number and diversity of organisms in DFUs compared to C&S methods.
- There was a low level of concordance between molecular methods and conventional culture techniques.

**How might this impact clinical practice in the foreseeable future?**

- Molecular microbiology has limited utility for mild or moderate DFUs but may aid in diagnosing infections in deep surgical sites or osteomyelitis, warranting further study.

## 1 INTRODUCTION

For individuals living with diabetes, foot ulcers are a major complication, affecting 2%–10% of this population annually.^1^, ^2^ The delayed healing of diabetic foot ulcers (DFUs) increases their susceptibility to new and recurrent infections. Without timely and effective treatment, these infections can lead to severe morbidity, including lower extremity amputation and mortality. Clinically, about 40% of DFUs are infected at the patient's initial presentation, underscoring the need for prompt and accurate diagnostic processes and appropriate antimicrobial therapy.^3^ Optimal management of DFU infections hinges on the rapid clinical identification of infection, and its severity, followed by microbiological investigations to determine the causative organisms and their antibiotic sensitivities.

The diagnosis of infection in DFUs is primarily based on clinical signs, such as erythema, warmth, induration, pain or tenderness, purulence or fever.^3^, ^4^, ^5^ Empiric antibiotic therapy should be initiated promptly in clinically infected DFUs. In the absence of microbiological data, initial antibiotic selection is guided by clinical presentation, history of infection, recent antimicrobial use and relevant prescribing guidelines. The escalating global challenge of antibiotic resistance has led to the development of antimicrobial stewardship (AMS) principles, which emphasises the judicious use of antibiotics. Prescribing the most appropriate, targeted antibiotic agents depends on refining therapy based upon identified pathogens and their antimicrobial susceptibilities.^5^, ^6^, ^7^ Empiric therapy plays a pivotal role in improving outcomes and limiting infection progression while awaiting microbiological results. Therapy can then be adjusted according to these findings to ensure optimal therapeutic efficacy.^7^

Microbiology investigations of infected wounds traditionally follow a culture‐based approach, involving the application of swabs or tissue samples onto appropriate agar media. These are used to cultivate clinically relevant pathogens for identification and to facilitate antimicrobial susceptibility testing of the cultured organisms. While these culture‐based methods have been used for decades and clearly support clinical management of infected DFUs, they have inherent limitations. These include the substantial time required to obtain results (typically at least 24–72 h) and the potential for missing fastidious or non‐culturable pathogens.^4^ The advent of molecular diagnostic techniques offers a faster and potentially more accurate means of identifying the microbial landscape of DFUs. These methods are, however, expensive, not widely available, and have not been clearly shown to improve clinical outcomes. Thus, their integration into clinical practice requires a thorough assessment to understand their agreement with conventional culture techniques and the implications of their use for patient management.

This substudy was conducted as part of the CODIFI2 trial (ISRCTN74929588), a Phase III, multi‐centre, prospective trial comparing swab versus tissue sampling in infected DFUs. It evaluates concordance between culture and sensitivity (C&S) and metagenomic 16S rRNA gene sequencing (M16S) in identifying microbial organisms in suspected infected DFUs. Paired samples from participants, with one processed by C&S and the other for M16S, were performed post hoc at a central laboratory. Reports generated by each method were then compared to assess concordance. This research aimed to assess the potential of a molecular microbiology approach to enhance DFU management by delivering a more comprehensive microbiology result. Earlier and more targeted antimicrobial interventions could improve patient outcomes and support AMS and contribute to reducing the global burden of antibiotic resistance.^8^, ^9^

This research could provide evidence supporting M16S as either an adjunct or an alternative to culture methods. Molecular approaches could, if shown to have good concordance with C&S and demonstrated to be reliable, cost‐effective with rapid results, become integrated into clinical pathways. Through a detailed comparison of these microbiological methodologies, this study provides valuable insights into their practical utility and impact on the clinical management of diabetic foot infections.

## 2 METHODS

CODIFI2 (ISRCTN74929588; registered on 8 January 2019) was a Phase III, multi‐centre, prospective, parallel group, randomised controlled trial. Its primary objective was to assess the clinical effectiveness of swab versus tissue sampling for microbiology investigations in individuals with suspected mild or moderate DFU infections. The primary outcome of the main trial was time to healing of the index ulcer. Within CODIFI2, this cross‐sectional substudy evaluated agreement between C&S and M16S in identifying key pathogens from paired samples collected at baseline clinical assessment.

### 2.1 Ethical approval

The study protocol was approved by the West of Scotland Research Ethics Committee (18/WS/0235), and informed consent was obtained from all participants. All investigations reported in this study adhered to the principles of the Declaration of Helsinki as revised in 2008.

### 2.2 Participants, setting and randomisation

Participants were recruited from 21 UK NHS secondary care and community clinics that offered multidisciplinary teams for DFU management. Eligible participants were individuals aged 18 years or older, attending a clinic for DFU, and with suspected mild or moderate soft tissue infection according to IDSA guidelines.^5^ Patients provided their consent on the day they visited the clinic with a suspected clinically infected DFU. Written consent was required for the study and foot photography. Patients were excluded if they had ulcers for more than 2 years, had suspected osteomyelitis or severe infection, were unwilling to comply with follow‐up or did not agree to have their sampling technique the randomly allocated way. Eligible and consenting participants were randomised to swab or tissue sampling with a minimisation algorithm incorporating a random element (80% chance of allocation reducing imbalances).

### 2.3 Interventions

Staff were required to clean and debride the wound prior to sampling. Participants were to have the index DFU (largest at baseline) and all other clinically infected DFUs sampled by the randomised method, with any deviations recorded. Swab samples were collected utilising the recommended Levine technique.^10^ As no such recommendation exists for tissue samples, these were collected using each clinic's preferred tissue sampling method. Methods included use of dermal curette, scalpel blade or tissue biopsy.

### 2.4 Sample collection

For this substudy, at baseline, a second paired sample was obtained by the randomised method for M16S analysis. Primary samples were forwarded to the respective local NHS accredited microbiology laboratories for C&S. The second paired sample was sent for molecular microbiology analysis, with reporting at trial end.

### 2.5 Samples for C&S testing

Samples were subjected to routine C&S testing, followed by reporting processes in accordance with locally established protocols. Data on identified organisms and their antibiotic sensitivities were systematically collected using case report forms. For ease of analysis and reporting, a coded list of the organisms was compiled, as detailed in Table 1.

**TABLE 1:** C&S reported organisms and corresponding code.

C&S code list	C&S reported organism
001	*Actinomyces* species
002	Anaerobic Gram‐negative cocci
003	Anaerobic Gram‐negative rods/bacilli
004	Anaerobic Gram‐positive rods/bacilli
005	Anaerobic Streptococci/cocci
006	Anaerobes (mixed)
007	*Bacteroides fragilis*/*B. fragilis*
008	*Bacteroides* species
009	Beta (β) haemolytic streptococci
010	*Corynebacterium diphtheria*
011	*Corynebacterium ulcerans*
012	*Candida* species
013	*Clostridium* species
014	CNS, Coagulase‐negative Staphylococcus/Coag‐neg staph
015	Coliform
016	*Corynebacterium* species
017	*Escherichia coli*
018	*Enterobacteriaceae*
019	*Enterobacter* species
020	*Enterococcus* species
021	Fungi
022	*Fusobacterium* species
023	*Finegoldia magna*
024	GAS/Group A Strep/Group A Streptococcus
025	GCS/Group C Strep/Group C Streptococcus
026	GGS/Group G Strep/Group G Streptococcus
027	*Klebsiella* species
028	Methicillin‐resistant *S. aureus* (MRSA)
029	*Morganella* species
030	*Peptococcus* species
031	*Peptoniphilus* species
032	*Peptostreptococcus* species
033	*Porphyromonas* species
034	*Prevotella* species
035	*Pseudomonas* species
036	*Staphylococcus aureus*
037	*Staphylococcus epidermidis*
038	Vancomycin‐resistant Enterococcus (VRE)
039	*Veillonella* species
040	Yeasts
041	*Acinetobacter* species
042	*Citrobacter* Species
043	Group B Strep/*S. agalactiae*/*Streptococcus agalactiae*
044	*Micrococcus* species
045	*Proteus* species
046	*Streptococcus Intermedius*

### 2.6 Samples for M16S molecular analysis

All swab and tissue samples for M16S analysis were taken in the same way as the respective paired sample for C&S investigations. For swabs, Copan flock swabs (FLOQSwabs) were used. Following collection, the tip of the swab or the tissue sample was preserved in a molecular sample collection tube as follows: Between 7 May 2019 and 19 November 2021, a total of 132 samples were preserved in Powersoil tubes (Qiagen DNeasy PowerSoil Kit). From 2 December 2021 to 29 April 2022, due to changes in the Powersoil tubes provided with the Qiagen DNeasy PowerSoil Kit, 15 samples were preserved using eNAT tubes (COPAN Diagnostics). The samples were refrigerated and dispatched to Nottingham University Hospitals NHS Trust within 48 h of being collected. Upon arrival, they were stored at −80°C until M16S batch processing and analysis.

### 2.7 Molecular sample processing and analysis

Detailed information on sample processing and analysis can be found in Appendix S1. Briefly, DNA was extracted from the samples before amplifying the V4 hypervariable region 16S rRNA gene using the prokaryote primers 515F and 806R.^11^ The amplified products were sequenced on an Illumina MiSeq platform using the 2 × 250 bp sequencing method.

### 2.8 Sequence preprocessing and taxonomic assignment

Analysis of sequencing data was conducted using QIIME2. Prokaryotic reads, once taxonomically assigned, were normalised to relative abundances by dividing the read counts by the total number of reads for each sample. Taxa identified were then aligned with a predefined shortlist of genera (a taxonomic rank above species) (Table 2), excluding any that did not match the list.

**TABLE 2:** Molecular genus and corresponding C&S codes, Gram classification and anaerobe status.

Corresponding C&S code	Molecular organism (genus)	Anaerobe Y/N	Gram classification positive (P) or negative (N)
001	*Actinomyces*	N	P
999	*Actinotignum*	N	P
999	*Arcanobacterium*	N	P
010 011 016	*Corynebacterium*	N	P
999	*Brevibacterium*	N	P
999	*Brachybacterium*	N	P
999	*Dermabacter*	N	P
044	*Micrococcaceae*.unclassified	N	P
999	*Kocuria*	N	P
044	*Micrococcus*	N	P
004	*Cutibacterium*	Y	P
007 008 003	*Bacteroides*	Y	N
003 033	*Porphyromonas*	Y	N
003 034	*Prevotella*	Y	N
999	*Capnocytophaga*	Y	N
999	*Sphingobacterium*	Y	N
999	*Campylobacter*	N	N
999	*Granulicatella*	N	P
020 038	*Enterococcus*	N	P
024 025 026 009 043 046	*Streptococcus*	N	P
999	*Gemella*	N	P
028 036 037 014	*Staphylococcus*	N	P
004	*Fastidiosipila*	Y	P
013 004	*Clostridium*.sensu.*stricto*.1	Y	P
030 005	*Peptococcus*	Y	P
032 005	*Peptostreptococcus*	Y	P
005	*Anaerococcus*	Y	P
005	*Ezakiella*	Y	P
023 005	*Finegoldia*	Y	P
005	*Helcococcus*	Y	P
005	*Parvimonas*	Y	P
031 005	*Peptoniphilus*	Y	P
002	*Dialister*	Y	N
039 002	*Veillonella*	Y	N
022 003	*Fusobacterium*	Y	N
999	*Alcaligenes*	N	N
999	*Neisseria*	N	N
015 018 019	*Enterobacter*	N	N
015 018 019 027 042 045	*Enterobacteriaceae*.unclassified	N	N
017 015 018	*Escherichia.Shigella*	N	N
015 018 027	*Klebsiella*	N	N
029 015	*Morganella*	N	N
015 045	*Proteus*	N	N
015	*Providencia*	N	N
015 018	*Serratia*	N	N
999	*Aggregatibacter*	N	N
999	*Haemophilus*	N	N
999	*Pasteurella*	N	N
041	*Acinetobacter*	N	N
999	*Moraxella*	N	N
035	*Pseudomonas*	N	N
999	*Stenotrophomonas*	N	N
012 021 040 999	Other	NA	

For comparison with culture data, a relative abundance threshold of 0.05 (5%) was established as significant for determining presence or absence, and any taxa below this threshold were omitted. The 0.05 relative abundance threshold was prespecified by the investigators at study outset, reflecting a commonly used threshold in microbiome studies to exclude low‐abundance taxa and reduce the influence of sequencing artefacts.^12^, ^13^, ^14^, ^15^ To ensure the relative abundance of each sample summed to one, a collective taxonomic category labelled ‘Other’ was introduced to aggregate all excluded abundances. All sequence data were submitted to NCBI under the BioProject number PRJNA 1119028.

### 2.9 Statistical analysis

The presence of a particular bacterial organism identified on the C&S microbiology report (Table 1) was mapped to corresponding genera from the molecular analysis via a predetermined list (Table 2). Specific named genera for the molecular analysis were derived as ‘present’ if the corresponding abundance of the pathogen was at least 0.05 for that named genus; otherwise, a result of ‘not present’ was derived.^12^ Concordance between the traditional C&S and molecular methods for detecting specific pathogen genera or classes in each paired DFU sample was evaluated using prevalence and agreement metrics. Cohen's Kappa, which corrects the simple agreement proportion for chance agreement that may occur when two raters act independently, was reported. The Prevalence and Bias Adjusted Kappa statistic (PABAK), which summarises agreement without being influenced by the genus prevalence or highly unbalanced presence ratings, was also included.

To further explore agreement patterns, multinomial univariable regression was performed, incorporating randomised allocation as a covariate. Difference in number of genera reported by molecular rather than C&S (i.e. 2+ fewer, 1 fewer, Same number, 1 more, 2 more, 3 + more) was analysed using ordinal univariable regression, with a covariate for randomised allocation.

## 3 RESULTS

Of the 147 participants, complete data were available from 145 for analysis. Data in Table 3 provide a summary of genera presence in baseline samples, extracted from the cross‐tabulations of C&S and M16S data (a complete table can be found in the Appendix S1). For C&S, no bacterial growth was reported in 37/145 samples (25.5%; 95% confidence interval: 18%–32.3%). In contrast, M16S detected ≥5% abundance of at least one target bacterium in all 145 samples (100%).

**TABLE 3:** Cross‐tabulations of reported presence of genus in baseline sample by C&S and molecular methods, with agreement statistics (Cohen's kappa with 95% CI, and PABAK).

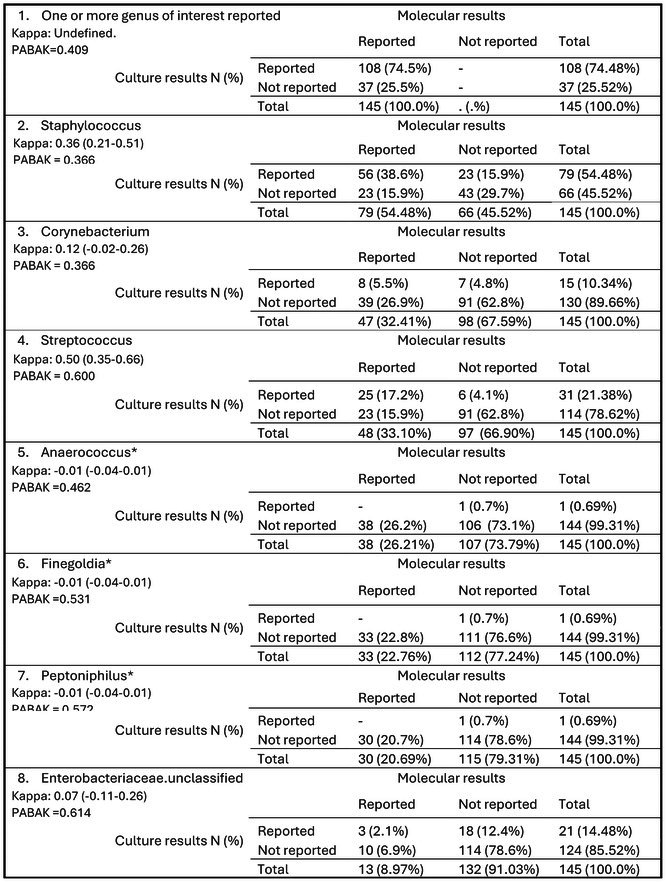

^a^ Denotes anaerobic organism.

The two most prevalent genera identified by both methods were *Staphylococcus* spp. and *Corynebacterium* spp. C&S identified *Staphylococcus* spp. in 79 samples, a number matched by M16S. However, the overlap in the samples with *Staphylococcus* spp. detected by both methods was not exact, with only 56 samples reporting these organisms in both approaches. For *Corynebacterium* spp., there was a notable difference in detection rates, with C&S methods reporting it in 15 samples, whereas molecular methods identified it in 47 samples, with agreement in eight samples (Table 3).

Summary data in Table 4 present a concise version of prevalence and agreement findings (a complete table can be found in the Appendix S1). Data show whether C&S/M16S reported the same bacterial genera, different genera or more genera. The M16S method reported a greater number and broader array of genera, reporting 455 genera across 40 distinct genera compared to 248 genera across 25 distinct genera reported by C&S.

**TABLE 4:** Summary of reported prevalence and agreement statistics for each specific genus of interest.

Genus	Reported present by either method	Reported present by molecular method	Reported present by C&S method	Difference in reported presence (95% CI) (molecular–C&S)	Agreement (%, 95% CI)	Disagreement (%, 95% CI)	Cohen's kappa (95% CI)	PABAK
One or more genus reported	145 (100.0%)	145 (100.0%)	108 (74.5%)	25.5% (18.0% to 32.3%)	74.5% (66.6–81.4)	25.5% (18.6–33.4)	–	0.409
Staphylococcus	102 (70.3%)	79 (54.5%)	79 (54.5%)	0.0% (−7.6% to 7.6%)	68.3% (60.0–75.7)	31.7% (24.3–40.0)	0.36 (0.21 to 0.51)	0.366
Corynebacterium	54 (37.2%)	47 (32.4%)	15 (10.3%)	22.1% (14.2% to 29.3%)	68.3% (60.0–75.7)	31.7% (24.3–40.0)	0.12 (−0.02 to 0.26)	0.366
Streptococcus	54 (37.2%)	48 (33.1%)	31 (21.4%)	11.7% (5.0% to 18.2%)	80.0% (72.6–86.2)	20.0% (13.8–27.4)	0.50 (0.35 to 0.66)	0.600
Anaerococcus^a^	39 (26.9%)	38 (26.2%)	1 (0.7%)	25.5% (17.9% to 32.4%)	73.1% (65.1–80.1)	26.9% (19.9–34.9)	−0.01 (−0.04 to 0.01)	0.462
Finegoldia^a^	34 (23.4%)	33 (22.8%)	1 (0.7%)	22.1% (14.8% to 28.7%)	76.6% (68.8–83.2)	23.4% (16.8–31.2)	−0.01 (−0.04 to 0.01)	0.531
Peptoniphilus^a^	31 (21.4%)	30 (20.7%)	1 (0.7%)	20.0% (13.0% to 26.5%)	78.6% (71.0–85.0)	21.4% (15.0–29.0)	−0.01 (−0.04 to 0.01)	0.572
Enterobacteriaceae.unclassified	31 (21.4%)	13 (9.0%)	21 (14.5%)	−5.5% (−12.0% to 1.1%)	80.7% (73.3–86.8)	19.3% (13.2–26.7)	0.07 (−0.11 to 0.26)	0.614
Prevotella^a^	15 (10.3%)	15 (10.3%)	–	10.3% (5.0% to 15.4%)	89.7% (83.5–94.1)	10.3% (5.9–16.5)	–	0.793
Enterococcus	15 (10.3%)	9 (6.2%)	9 (6.2%)	0.0% (−4.7% to 4.7%)	91.7% (86.0–95.7)	8.3% (4.3–14.0)	0.29 (−0.00 to 0.58)	0.834
Pseudomonas	14 (9.7%)	12 (8.3%)	10 (6.9%)	1.4% (−2.3% to 5.0%)	95.9% (91.2–98.5)	4.1% (1.5–8.8)	0.71 (0.48 to 0.93)	0.917
Porphyromonas^a^	12 (8.3%)	12 (8.3%)	–	8.3% (3.4% to 12.9%)	91.7% (86.0–95.7)	8.3% (4.3–14.0)	–	0.834
Klebsiella	12 (8.3%)	–	12 (8.3%)	−8.3% (−12.9% to −3.4%)	91.7% (86.0–95.7)	8.3% (4.3–14.0)	–	0.834
Cutibacterium^a^	7 (4.8%)	7 (4.8%)	–	4.8% (0.9% to 8.6%)	95.2% (90.3–98.0)	4.8% (2.0–9.7)	–	0.903
Veillonella^a^	6 (4.1%)	6 (4.1%)	–	4.1% (0.4% to 7.7%)	95.9% (91.2–98.5)	4.1% (1.5–8.8)	–	0.917
Fusobacterium^a^	6 (4.1%)	6 (4.1%)	–	4.1% (0.4% to 7.7%)	95.9% (91.2–98.5)	4.1% (1.5–8.8)	–	0.917
Bacteroides^a^	4 (2.8%)	4 (2.8%)	–	2.8% (−0.5% to 5.9%)	97.2% (93.1–99.2)	2.8% (0.8–6.9)	–	0.945
Ezakiella^a^	2 (1.4%)	1 (0.7%)	1 (0.7%)	0.0% (−2.6% to 2.6%)	98.6% (95.1–99.8)	1.4% (0.2–4.9)	−0.01 (−0.02 to 0.00)	0.972
Dialister^a^	1 (0.7%)	1 (0.7%)	–	0.7% (−1.6% to 3.0%)	99.3% (96.2–100.0)	0.7% (0.0–3.8)	–	0.986

^a^ Anaerobic organism.

Overall, 11 genera were found to have a prevalence of 10% or higher, as detected by either analysis method (Table 4). Data show a higher detection rate for specific named bacterial genera in molecular analyses compared to C&S techniques. M16S identified seven genera with a prevalence of 10% or higher. The rank order of these were *Staphylococcus* > *Corynebacterium* > *Streptococcus > Anaerococcus > Finegoldia > Peptoniphilus* > *Prevotella*. Meanwhile, C&S identified four genera with a prevalence of 10% or higher, ranked as *Staphylococcus > Corynebacterium > Streptococcus > Enterobacteriaceae*.

The M16S method detected a higher number and wider variety of anaerobic genera compared to C&S. M16S identified a total of 173 anaerobic organisms across 15 distinct genera, whereas C&S reported eight distinct anaerobic genera. Specifically, M16S found *Anaerococcus* spp. in 26.2% of samples, *Finegoldia* spp. in 22.8% and *Peptoniphilus* spp. in 20.7%, whereas C&S detected these genera in only 0.7% of samples. Additionally, M16S identified *Prevotella* spp., *Porphyromonas* spp., *Veillonella* spp., *Fusobacterium* spp., *Cutibacterium* spp., *Bacteroides* spp., *Dialister* spp. and *Ezakiella* spp. none of which were reported in any samples by C&S.

Overall, there were high levels of observed agreement on the presence or absence of bacteria between the C&S and M16S, with over 75% agreement for the majority of genera. This high rate of agreement was particularly evident in cases where organisms were reported absent by both methods. However, when adjusting for chance agreement using Cohen's Kappa, the overall concordance between C&S and M16S molecular method was found to be low. Only *Pseudomonas* spp. and *Streptococcus* spp. demonstrated a Kappa value of 0.5 or higher. For many genera, Kappa values were close to or below zero, suggesting agreement worse than would be expected by chance. Furthermore, the PABAK values exceeded the corresponding Kappa values across all evaluated genera, underscoring the significant impact of genus prevalence on the observed levels of agreement. This adjustment suggests that the percentage agreement metrics might be unduly influenced by the uneven distribution of specific genera within the sample population. In cases involving the most prevalent genera, discrepancies were frequently observed between C&S and M16S molecular method regarding the detection of the genus. The M16S analyses were more consistently inclined to identify the presence of a genus compared to C&S reports, reflecting a systematic tendency towards higher detection rates by the M16S approach.

## 4 DISCUSSION

This study investigates the concordance between traditional C&S methods and metagenomic 16S rRNA (M16S) gene sequencing molecular method in identifying microbial organisms in infected DFUs. Given the importance of timely and accurate pathogen detection, this study assessed the potential of metagenomic 16S sequencing to support the management of mild to moderate DFU infections.

The 16S V4 hypervariable region and Illumina platform was selected for their established performance in profiling diverse microbial communities, offering a balance between sequencing depth and taxonomic resolution. Compared to C&S, this approach detected a higher number of organisms across a broader range of genera (455 across 40 genera, including 173 anaerobes across 15 genera), while C&S identified 248 organisms across 25 genera, with 8 individual anaerobic genera. Despite a high level of observed agreement, adjustments for chance agreement using Cohen's Kappa, indicated a low concordance.

Targeting the 16S V4 hypervariable region enabled broad microbial profiling, allowing characterisation of complex communities and detection of organisms potentially relevant to wound infections.^16^ However, this molecular strategy has inherent limitations, notably in the taxonomic resolution of 16S amplicon sequences, considered accurate only to the family or genus level. This limitation is evident when distinguishing closely related groups like *Enterobacteriaceae*.^17^ Considering these limitations a pragmatic approach was adopted: reported C&S organisms were mapped back to their genus level, enabling a realistic and practical comparison between C&S and M16S. This ensured that the utility of this molecular approach could be explored relative to clinical practice pathways. A key strength is the use of authentic C&S reports from accredited NHS microbiology laboratories, grounding the findings in real‐world clinical practice rather than research settings, which differ in resources, time constraints, and diagnostic priorities. A critical aspect of clinical microbiology laboratories is the interpretation of cultures, focussing on likely pathogens while enabling timely reporting to guide therapy.^16^ This means that not all cultured organisms are identified or included in the report, as clinical relevance and urgency in initiating antimicrobial therapy guide reporting decisions. The discrepancy in reported organisms between methods may reflect C&S reporting practices, combined with the greater sensitivity of M16S in detecting low‐abundance or fastidious organisms. Namely, C&S reports synthesise laboratory results with clinical judgement, identifying and reporting only organisms and susceptibilities deemed most relevant for patient management.

Molecular analysis of infected DFU samples is not yet routine in clinical microbiology laboratories, limiting expert microbiology input and standardised reporting. Organisms with a relative abundance >0.05 were reported, reflecting a common threshold in 16S Illumina sequencing to reduce artefacts and improve data quality.^12^ This approach minimises spurious taxa that arise from sequencing errors or cross‐contamination.^13^, ^14^, ^15^ However, it may exclude clinically relevant organisms present at lower abundances, particularly those that are suppressed by dominant taxa, or not efficiently amplified during PCR.^18^ The threshold represents a trade‐off between analytical robustness and clinical sensitivity. Our M16S data strongly aligns with previous reports on DFU microbiota, particularly showing enhanced levels of *Staphylococcus* spp., *Corynebacterium* spp. and anaerobes.^11^, ^19^, ^20^, ^21^ Our data also show a high level of observed agreement between C&S and M16S, consistent with other agreement studies^22^, ^23^ however, concordance after adjustments for chance was generally low.

Adjusting for chance agreement with Cohen's Kappa is essential, as it reveals true concordance between the methods beyond chance. This low concordance likely arises from fundamental differences in how organisms are reported. Metagenomic 16 s methods will indiscriminately identify all organisms present, providing they have sufficient, intact target DNA and are above the 5% threshold. By contrast, clinical microbiology laboratories selectively report organisms based on clinical relevance, often omitting those deemed insignificant or grouping them under general categories such as ‘skin flora’. This distinction is illustrated by *Staphylococcus* spp. M16S may detect both *Staphylococcus aureus*, a key DFU pathogen, and coagulase‐negative *Staphylococci* (CNS), which are skin commensals. However, both would be reported under genus *Staphylococcus* spp., making it difficult to distinguish their clinical relevance from genus‐level data alone. In contrast, C&S reports typically prioritise reporting of *S. aureus* and may disregard or report CNS under non‐specific categories. Similar discrepancies may extend to other organisms like *Corynebacteria* spp. and enteric flora, where clinical judgement informs selective reporting. Access to bacterial identification tools across laboratories, such as mass spectrometry and local variations in laboratory protocols may also influence the number of organisms reported in C&S.

The paired samples for molecular analysis were collected from the same wound at the same visit, using the same method. Tissue samples were obtained according to local practice, using techniques such as dermal curette, scalpel blade, or biopsy. These approaches can differ in terms of the depth and volume of tissue collected, which may have influenced microbial yield, and the types of organisms detected. Unlike wound swabs, where the Levine technique is recommended, there is no standardised method for obtaining tissue samples from DFUs. It is possible that some techniques may favour recovery of viable organisms for culture, while others may be more suited to preserving microbial DNA for sequencing. The extent to which tissue sampling technique affects the comparative performance of diagnostic methods is unclear and may have contributed to variability in detection. This remains an important area for further research.

Importantly, discrepancies were not one‐sided. Despite the enhanced sensitivity of M16S, C&S occasionally reported taxa absent from the paired molecular analysis. This may be due to the sampling order, with molecular swabs collected after C&S, potentially depleting biomass. Limited taxonomic resolution in M16S, along with differences in tissue sampling techniques that may favour one diagnostic approach over the other, could also have contributed. Overall, these findings highlight that neither approach offered greater utility in identifying organisms in samples.

Despite the low level of concordance, M16S was notable for its detection of a greater number and a wider variety of organisms, including anaerobes. In this study, 25.5% (95% CI: 18.0%–32.3%) of samples processed through C&S methods reported no bacterial growth, whereas M16S identified at least one organism above the 5% abundance threshold in all 145 samples. This suggests a greater sensitivity of M16S in detecting organisms that may be missed by culture, particularly those present at low abundance or that are difficult to culture, such as anaerobes. The clinical significance of identifying this broader range of taxa in DFUs is unclear. It is not known whether these represent colonisers or organisms with a pathogenic role. In particular, the role of low abundant anaerobes in DFU is not well defined. If colonising anaerobes contribute to infection pathogenesis, their underrepresentation in culture‐based methods could lead to their exclusion from treatment considerations, potentially impacting patient outcomes. Conversely, if they are not clinically relevant, their detection may have limited therapeutic implications. This uncertainty raises concerns about overinterpretation of sequencing data, including unnecessary antibiotic use. Conventional culture methods may already capture the most clinically significant pathogens, those most likely to benefit from targeted therapy. This ambiguity highlights the ongoing challenge of differentiating colonisation from infection when tailoring effective treatment strategies for DFUs.

Greater sensitivity of molecular approaches may be of utility in samples where enrichment or extended culture is recommended to recover fastidious or low abundance organisms, such as bone and soft tissue infections.^24^ Osteomyelitis is a common complication in DFUs. While M16S may have utility in this context, patients with suspected osteomyelitis were excluded from this study and thus its application in this group is beyond its scope. Further research is warranted to explore M16S potential role in such settings.

The practical implications of implementing M16S in routine clinical microbiology were not examined in this study but are essential to consider when evaluating its potential utility. In addition to the low concordance observed, several barriers limit its feasibility in real‐world settings, including higher costs, the need for trained personnel and the ability to perform timely bioinformatic analysis. Although sequencing may be completed within 24–48 h, downstream bioinformatic analysis and reporting can extend turnaround times beyond what is practical for time‐sensitive infection management. Interpretation of sequencing data remains challenging, particularly in distinguishing clinically significant pathogens from colonising flora, and without accompanying antibiotic susceptibility data, the results may not be readily actionable. Taken together, these considerations currently limit the clinical applicability of M16S for DFU management.

## 5 CONCLUSIONS

This study examined the concordance between standard C&S and M16S molecular methods to evaluate M16S as an adjunct or alternative to traditional culture techniques. The M16S molecular method, which can detect low abundance or fastidious organisms and provide expedited results, have the potential to influence clinical pathways and outcomes in DFU management. Despite identifying a broader range of organisms, particularly fastidious anaerobes, the M16S showed low concordance with C&S. Our findings suggest that traditional culture techniques might not fully capture the diversity and prevalence of anaerobes in DFUs. Yet, the clinical significance of these anaerobes and other organisms, identified through M16S, remains poorly understood. Molecular approaches may offer advantages in infections where increased sensitivity in microbial detection is beneficial, particularly in cases where extended culture techniques are required to detect fastidious or low‐abundance organisms. These findings warrant further investigation into the clinical utility of such methods in settings where conventional culture may be limited.

Diagnostic microbiology laboratories play a crucial role in interpreting the relevance of microbiology findings, essential to prevent antimicrobial overprescription and ensure effective infection management, especially since most molecular microbiology methods lack antibiotic sensitivity data. Of note, some organisms were detected by C&S but not by the M16S molecular technique; hence neither approach was more sensitive for every sample.

Incorporating a molecular microbiology approach into clinical practice would not only involve economic considerations but also require clinical guidance for interpreting molecular reports, especially if C&S were retained, with molecular approaches as an adjunct. The findings of this study suggest that M16S may not yet be a replacement for C&S. It is notable that it might instead be considered as complementary to C&S, and this has been further explored in a companion qualitative study.^25^ For mild to moderate infections, this study supports continued reliance on conventional C&S testing due to its proven ability to identify DFU pathogens and generate antibiotic sensitivity data. Its ongoing clinical value is reinforced by established interpretative frameworks and microbiological expertise, enabling timely and appropriate antimicrobial therapy.

## FUNDING INFORMATION

This study was funded by the UK National Institute for Health Research Health Technology Assessment Programme. It was a commissioned call for research (reference 16/163/04); hence, the funders specified the study population and sample, the intervention and the primary outcomes. The sponsor was the University of Leeds. The sponsor and funder were not involved in the design of the study; the collection, analysis and interpretation of data; writing the report and did not impose any restrictions regarding the publication of the report.

## CONFLICT OF INTEREST STATEMENT

None.

## Supporting information


Appendix S1

## Data Availability

All sequence data were submitted to NCBI under the BioProject number PRJNA 1119028. Further data supporting the results of this article can be found in the NIHR synopsis.
