# Supplementary material for: Concordance of molecular microbiology and conventional culture techniques for infected diabetic foot ulcer management
Source: Diabet Med. 2025 Jul 9;42(9):e70089. doi: 10.1111/dme.70089 (PMC12352717; doi:10.1111/dme.70089)
Supplement: Supplementary file 1 — Appendix S1 [file DME-42-e70089-s001.docx]

**Supplementary Materials**

**Concordance of Molecular Microbiology and Conventional Culture Techniques for Infected Diabetic Foot Ulcer Management**

**Molecular Analysis Methods:**

***DNA Extraction and Quantification***

DNA from samples in Powersoil tubes underwent extraction utilising the Qiagen DNeasy PowerSoil Kit following the manufacturer's instructions, incorporating an initial homogenisation step at 4500 rpm for 45 seconds, repeated three times. For samples in eNAT tubes, the extraction process employed the Qiagen DNeasy PowerSoil Pro Kit, adhering to the manufacturer's pro to col with minor adjustments. Swab tips or tissue samples were sterilely placed into PowerBead Pro Tubes, and after adding Solution CD1, samples were homogenised at the same speed and duration before continuation.

The extracted DNA was then s to red at -80 °C for future analyses. DNA quantification was carried out in duplicate using the Quant-iT PicoGreen dsDNA Assay Kit (Invitrogen) and measurements were taken twice on an ABI7500 system (Applied Biosystems). Additionally, DNA quality was assessed using the NanoDrop One device (Thermo Scientific), with a mean A260/280 ratio of 1.63 (SD = 0.54), median A260/280 ratio of 1.81 (n = 145).

***16S rRNA gene amplification, library preparation and sequencing***

The V4 hypervariable region 16SrRNA gene was amplified using the prokaryote primers^1^;

515F:TCGTCGGCAGCGTCAGATGTGTATAAGAGACAGGTGCCAGCMGCCGCGGTAA

806R:GTCTCGTGGGCTCGGAGATGTGTATAAGAGACAGGGACTACHVGGGTWTCTAAT

PCR amplification was performed using Phusion Hot Start II High-Fidelity PCR Master Mix (Thermo Scientific), with a final primer concentration of 0.2 µM. touch-down PCR was employed to reduce non-specific binding. Cycling conditions were as follows: one cycle at 95 °C for 3 min; 10 cycles of 95 °C for 30s, 65 °C to 55 °C (<1 °C every cycle) for 30 s, 72 °C for 30s; followed by 15 cycles of 95 °C for 30s, 55 °C for 30 s, 72 °C for 30 s; and a final cycle of 72 °C for 5 min.

PCR products were purified using AMPure XP beads (Beckman Coulter). In accordance with Illumina's protocols, each sample was tagged with barcodes and Illumina sequencing adapters utilising the Nextera XT Index Kit. These barcoded samples were then combined and sequenced on an Illumina MiSeq platform using the 2×250 bp sequencing method. The resulting FASTQ files were processed for taxonomic classification.

***Sequence pre-processing and taxonomic assignment***

Analysis of sequencing data was conducted using QIIME2. The initial set of samples, numbered 00001 to 00078, was processed with QIIME2-2020.11, while samples 00079 to 000149 utilised QIIME2-2022.8 for analysis. The procedure involved filtering, trimming, and dereplicating the raw data via DADA2, from which amplicon sequence variants (ASVs) were derived. These ASVs were then taxonomically categorised using a pre-trained Naïve Bayes Silva classifier within the 138 99% OTU database^2,3^. In the initial phase of filtering, ASVs identified as Eukaryota were excluded.

Table 3. Cross-tabulations of reported presence of genus in baseline sample by C&S and molecular methods, with agreement statistics (Cohen’s kappa with 95% CI, and PABAK). * Denotes anaerobic organism.

| 1. One or more genus of interest reported |  | Molecular results | |  |
| --- | --- | --- | --- | --- |
| Kappa: Undefined.  PABAK=0.409 |  | Reported | Not reported | Total |
| Culture results N (%) | Reported | 108 (74.5%) | - | 108 (74.48%) |
|  | Not reported | 37 (25.5%) | - | 37 (25.52%) |
|  | Total | 145 (100.0%) | . (.%) | 145 (100.0%) |
| 1. Staphylococcus   Kappa: 0.36 (0.21-0.51)  PABAK = 0.366 |  | Molecular results | |  |
|  |  | Reported | Not reported | Total |
| Culture results N (%) | Reported | 56 (38.6%) | 23 (15.9%) | 79 (54.48%) |
|  | Not reported | 23 (15.9%) | 43 (29.7%) | 66 (45.52%) |
|  | Total | 79 (54.48%) | 66 (45.52%) | 145 (100.0%) |
| 1. Corynebacterium |  | Molecular results | |  |
| Kappa: 0.12 (-0.02-0.26)  PABAK = 0.366 |  | Reported | Not reported | Total |
| Culture results N (%) | Reported | 8 (5.5%) | 7 (4.8%) | 15 (10.34%) |
|  | Not reported | 39 (26.9%) | 91 (62.8%) | 130 (89.66%) |
|  | Total | 47 (32.41%) | 98 (67.59%) | 145 (100.0%) |
| 1. Streptococcus |  | Molecular results | |  |
| Kappa: 0.50 (0.35-0.66)  PABAK = 0.600 |  | Reported | Not reported | Total |
| Culture results N (%) | Reported | 25 (17.2%) | 6 (4.1%) | 31 (21.38%) |
|  | Not reported | 23 (15.9%) | 91 (62.8%) | 114 (78.62%) |
|  | Total | 48 (33.10%) | 97 (66.90%) | 145 (100.0%) |
| 1. Anaerococcus* |  | Molecular results | |  |
| Kappa: -0.01 (-0.04-0.01)  PABAK =0.462 |  | Reported | Not reported | Total |
| Culture results N (%) | Reported | - | 1 (0.7%) | 1 (0.69%) |
|  | Not reported | 38 (26.2%) | 106 (73.1%) | 144 (99.31%) |
|  | Total | 38 (26.21%) | 107 (73.79%) | 145 (100.0%) |
| 1. Finegoldia* |  | Molecular results | |  |
| Kappa: -0.01 (-0.04-0.01)  PABAK =0.531 |  | Reported | Not reported | Total |
| Culture results N (%) | Reported | - | 1 (0.7%) | 1 (0.69%) |
|  | Not reported | 33 (22.8%) | 111 (76.6%) | 144 (99.31%) |
|  | Total | 33 (22.76%) | 112 (77.24%) | 145 (100.0%) |
| 1. Peptoniphilus* |  | Molecular results | |  |
| Kappa: -0.01 (-0.04-0.01)  PABAK = 0.572 |  | Reported | Not reported | Total |
| Culture results N (%) | Reported | - | 1 (0.7%) | 1 (0.69%) |
|  | Not reported | 30 (20.7%) | 114 (78.6%) | 144 (99.31%) |
|  | Total | 30 (20.69%) | 115 (79.31%) | 145 (100.0%) |
| 1. Enterobacteriaceae.unclassified |  | Molecular results | |  |
| Kappa: 0.07 (-0.11-0.26)  PABAK =0.614 |  | Reported | Not reported | Total |
| Culture results N (%) | Reported | 3 (2.1%) | 18 (12.4%) | 21 (14.48%) |
|  | Not reported | 10 (6.9%) | 114 (78.6%) | 124 (85.52%) |
|  | Total | 13 (8.97%) | 132 (91.03%) | 145 (100.0%) |
| 1. Escherichia.Shigella |  | Molecular results | |  |
| Kappa: 0.34 (0.09-0.60)  PABAK = 0.793 |  | Reported | Not reported | Total |
| Culture results N (%) | Reported | 5 (3.4%) | 7 (4.8%) | 12 (8.28%) |
|  | Not reported | 8 (5.5%) | 125 (86.2%) | 133 (91.72%) |
|  | Total | 13 (8.97%) | 132 (91.03%) | 145 (100.0%) |
| 1. Proteus |  | Molecular results | |  |
| Kappa: 0.43 (0.18-0.69)  PABAK = 0.821 |  | Reported | Not reported | Total |
| Culture results N (%) | Reported | 6 (4.1%) | 8 (5.5%) | 14 (9.66%) |
|  | Not reported | 5 (3.4%) | 126 (86.9%) | 131 (90.34%) |
|  | Total | 11 (7.59%) | 134 (92.41%) | 145 (100.0%) |
| 1. Prevotella* |  | Molecular results | |  |
| Kappa: Undefined  PABAK = 0.793 |  | Reported | Not reported | Total |
| Culture results N (%) | Reported | - | - | - |
|  | Not reported | 15 (10.3%) | 130 (89.7%) | 145 (100.0%) |
|  | Total | 15 (10.34%) | 130 (89.66%) | 145 (100.0%) |
| 1. Enterococcus |  | Molecular results | |  |
| Kappa: 0.29 (-0.00-0.58)  PABAK = 0.834 |  | Reported | Not reported | Total |
| Culture results N (%) | Reported | 3 (2.1%) | 6 (4.1%) | 9 (6.21%) |
|  | Not reported | 6 (4.1%) | 130 (89.7%) | 136 (93.79%) |
|  | Total | 9 (6.21%) | 136 (93.79%) | 145 (100.0%) |
| 1. Pseudomonas |  | Molecular results | |  |
| Kappa: 0.71 (0.48-0.93)  PABAK = 0.917 |  | Reported | Not reported | Total |
| Culture results N (%) | Reported | 8 (5.5%) | 2 (1.4%) | 10 (6.90%) |
|  | Not reported | 4 (2.8%) | 131 (90.3%) | 135 (93.10%) |
|  | Total | 12 (8.28%) | 133 (91.72%) | 145 (100.0%) |
| 1. Porphyromonas* |  | Molecular results | |  |
| Kappa: Undefined  PABAK = 0.834 |  | Reported | Not reported | Total |
| Culture results N (%) | Reported | - | - | - |
|  | Not reported | 12 (8.3%) | 133 (91.7%) | 145 (100.0%) |
|  | Total | 12 (8.28%) | 133 (91.72%) | 145 (100.0%) |
| 1. Klebsiella |  | Molecular results | |  |
| Kappa: Undefined  PABAK = 0.834 |  | Reported | Not reported | Total |
| Culture results N (%) | Reported | - | 12 (8.3%) | 12 (8.28%) |
|  | Not reported | - | 133 (91.7%) | 133 (91.72%) |
|  | Total | . (.%) | 145 (100.0%) | 145 (100.0%) |
| 1. Helcococcus* |  | Molecular results | |  |
| Kappa: -0.01 (-0.04-0.01)  PABAK = 0.848 |  | Reported | Not reported | Total |
| Culture results N (%) | Reported | - | 1 (0.7%) | 1 (0.69%) |
|  | Not reported | 10 (6.9%) | 134 (92.4%) | 144 (99.31%) |
|  | Total | 10 (6.90%) | 135 (93.10%) | 145 (100.0%) |
| 1. Morganella |  | Molecular results | |  |
| Kappa: 0.13 (-0.16-0.42)  PABAK = 0.862 |  | Reported | Not reported | Total |
| Culture results N (%) | Reported | 1 (0.7%) | 6 (4.1%) | 7 (4.83%) |
|  | Not reported | 4 (2.8%) | 134 (92.4%) | 138 (95.17%) |
|  | Total | 5 (3.45%) | 140 (96.55%) | 145 (100.0%) |
| 1. Enterobacter |  |  | |  |
| Kappa: Undefined  PABAK = 0.862 |  | Reported | Not reported | Total |
| Culture results N (%) | Reported | - | 10 (6.9%) | 10 (6.90%) |
|  | Not reported | - | 135 (93.1%) | 135 (93.10%) |
|  | Total | . (.%) | 145 (100.0%) | 145 (100.0%) |
| 1. Alcaligenes |  | Molecular results | |  |
| Kappa: Undefined  PABAK = 0.876 |  | Reported | Not reported | Total |
| Culture results N (%) | Reported | - | - | - |
|  | Not reported | 9 (6.2%) | 136 (93.8%) | 145 (100.0%) |
|  | Total | 9 (6.21%) | 136 (93.79%) | 145 (100.0%) |
| 1. Providencia* |  | Molecular results | |  |
| Kappa: -0.02 (-0.05-0.00)  PABAK = 0.876 |  | Reported | Not reported | Total |
| Culture results N (%) | Reported | - | 7 (4.8%) | 7 (4.83%) |
|  | Not reported | 2 (1.4%) | 136 (93.8%) | 138 (95.17%) |
|  | Total | 2 (1.38%) | 143 (98.62%) | 145 (100.0%) |
| 1. Serratia |  | Molecular results | |  |
| Kappa: -0.01 (-0.03-0.01)  PABAK =0.876 |  | Reported | Not reported | Total |
| Culture results N (%) | Reported | - | 8 (5.5%) | 8 (5.52%) |
|  | Not reported | 1 (0.7%) | 136 (93.8%) | 137 (94.48%) |
|  | Total | 1 (0.69%) | 144 (99.31%) | 145 (100.0%) |
| 1. Haemophilus |  | Molecular results | |  |
| Kappa: Undefined  PABAK = 0.876 |  | Reported | Not reported | Total |
| Culture results N (%) | Reported | - | - | - |
|  | Not reported | 9 (6.2%) | 136 (93.8%) | 145 (100.0%) |
|  | Total | 9 (6.21%) | 136 (93.79%) | 145 (100.0%) |
| 1. Peptostreptococcus* |  | Molecular results | |  |
| Kappa: -0.01 (-0.03-0.01)  PABAK = 0.890 |  | Reported | Not reported | Total |
| Culture results N (%) | Reported | - | 1 (0.7%) | 1 (0.69%) |
|  | Not reported | 7 (4.8%) | 137 (94.5%) | 144 (99.31%) |
|  | Total | 7 (4.83%) | 138 (95.17%) | 145 (100.0%) |
| 1. Cutibacterium* |  | Molecular results | |  |
| Kappa: Undefined  PABAK = 0.903 |  | Reported | Not reported | Total |
| Culture results N (%) | Reported | - | - | - |
|  | Not reported | 7 (4.8%) | 138 (95.2%) | 145 (100.0%) |
|  | Total | 7 (4.83%) | 138 (95.17%) | 145 (100.0%) |
| 1. Veillonella* |  | Molecular results | |  |
| Kappa: Undefined  PABAK = 0.917 |  | Reported | Not reported | Total |
| Culture results N (%) | Reported | - | - | - |
|  | Not reported | 6 (4.1%) | 139 (95.9%) | 145 (100.0%) |
|  | Total | 6 (4.14%) | 139 (95.86%) | 145 (100.0%) |
| 1. Fusobacterium* |  | Molecular results | |  |
| Kappa: Undefined  PABAK = 0.917 |  | Reported | Not reported | Total |
| Culture results N (%) | Reported | - | - | - |
|  | Not reported | 6 (4.1%) | 139 (95.9%) | 145 (100.0%) |
|  | Total | 6 (4.14%) | 139 (95.86%) | 145 (100.0%) |
| 1. Acinetobacter |  | Molecular results | |  |
| Kappa: -0.01 (-0.03-0.01)  PABAK = 0.917 |  | Reported | Not reported | Total |
| Culture results N (%) | Reported | - | 1 (0.7%) | 1 (0.69%) |
|  | Not reported | 5 (3.4%) | 139 (95.9%) | 144 (99.31%) |
|  | Total | 5 (3.45%) | 140 (96.55%) | 145 (100.0%) |
| 1. Actinomyces |  | Molecular results | |  |
| Kappa: -0.02 (-0.03-0.00)  PABAK = 0.931 |  | Reported | Not reported | Total |
| Culture results N (%) | Reported | - | 2 (1.4%) | 2 (1.38%) |
|  | Not reported | 3 (2.1%) | 140 (96.6%) | 143 (98.62%) |
|  | Total | 3 (2.07%) | 142 (97.93%) | 145 (100.0%) |
| 1. Bacteroides* |  | Molecular results | |  |
| Kappa: Undefined  PABAK = 0.945 |  | Reported | Not reported | Total |
| Culture results N (%) | Reported | - | - | - |
|  | Not reported | 4 (2.8%) | 141 (97.2%) | 145 (100.0%) |
|  | Total | 4 (2.76%) | 141 (97.24%) | 145 (100.0%) |
| 1. Brevibacterium |  | Molecular results | |  |
| Kappa: Undefined  PABAK = 0.959 |  | Reported | Not reported | Total |
| Culture results N (%) | Reported | - | - | - |
|  | Not reported | 3 (2.1%) | 142 (97.9%) | 145 (100.0%) |
|  | Total | 3 (2.07%) | 142 (97.93%) | 145 (100.0%) |
| 1. Micrococcaceae.unclassified |  | Molecular results | |  |
| Kappa: -0.01 (-0.02-0.00)  PABAK = 0.959 |  | Reported | Not reported | Total |
| Culture results N (%) | Reported | - | 1 (0.7%) | 1 (0.69%) |
|  | Not reported | 2 (1.4%) | 142 (97.9%) | 144 (99.31%) |
|  | Total | 2 (1.38%) | 143 (98.62%) | 145 (100.0%) |
| 1. Parvimonas* |  | Molecular results | |  |
| Kappa: -0.01 (-0.02-0.00)  PABAK = 0.959 |  | Reported | Not reported | Total |
| Culture results N (%) | Reported | - | 1 (0.7%) | 1 (0.69%) |
|  | Not reported | 2 (1.4%) | 142 (97.9%) | 144 (99.31%) |
|  | Total | 2 (1.38%) | 143 (98.62%) | 145 (100.0%) |
| 1. Micrococcus |  | Molecular results | |  |
| Kappa: -0.01 (-0.02-0.00)  PABAK = 0.972 |  | Reported | Not reported | Total |
| Culture results N (%) | Reported | - | 1 (0.7%) | 1 (0.69%) |
|  | Not reported | 1 (0.7%) | 143 (98.6%) | 144 (99.31%) |
|  | Total | 1 (0.69%) | 144 (99.31%) | 145 (100.0%) |
| 1. Campylobacter |  | Molecular results | |  |
| Kappa: Undefined  PABAK =0.972 |  | Reported | Not reported | Total |
| Culture results N (%) | Reported | - | - | - |
|  | Not reported | 2 (1.4%) | 143 (98.6%) | 145 (100.0%) |
|  | Total | 2 (1.38%) | 143 (98.62%) | 145 (100.0%) |
| 1. Gemella |  | Molecular results | |  |
| Kappa: Undefined  PABAK =0.972 |  | Reported | Not reported | Total |
| Culture results N (%) | Reported | - | - | - |
|  | Not reported | 2 (1.4%) | 143 (98.6%) | 145 (100.0%) |
|  | Total | 2 (1.38%) | 143 (98.62%) | 145 (100.0%) |
| 1. Ezakiella* |  | Molecular results | |  |
| Kappa: -0.01 (-0.02-0.00)  PABAK = 0.972 |  | Reported | Not reported | Total |
| Culture results N (%) | Reported | - | 1 (0.7%) | 1 (0.69%) |
|  | Not reported | 1 (0.7%) | 143 (98.6%) | 144 (99.31%) |
|  | Total | 1 (0.69%) | 144 (99.31%) | 145 (100.0%) |
| 1. Pasteurella |  | Molecular results | |  |
| Kappa: Undefined  PABAK = 0.972 |  | Reported | Not reported | Total |
| Culture results N (%) | Reported | - | - | - |
|  | Not reported | 2 (1.4%) | 143 (98.6%) | 145 (100.0%) |
|  | Total | 2 (1.38%) | 143 (98.62%) | 145 (100.0%) |
| 1. Actinotignum |  | Molecular results | |  |
| Kappa: Undefined  PABAK = 0.986 |  | Reported | Not reported | Total |
| Culture results N (%) | Reported | - | - | - |
|  | Not reported | 1 (0.7%) | 144 (99.3%) | 145 (100.0%) |
|  | Total | 1 (0.69%) | 144 (99.31%) | 145 (100.0%) |
| 1. Arcanobacterium |  | Molecular results | |  |
| Kappa: Undefined  PABAK = 0.986 |  | Reported | Not reported | Total |
| Culture results N (%) | Reported | - | - | - |
|  | Not reported | 1 (0.7%) | 144 (99.3%) | 145 (100.0%) |
|  | Total | 1 (0.69%) | 144 (99.31%) | 145 (100.0%) |
| 1. Fastidiosipila* |  | Molecular results | |  |
| Kappa: Undefined  PABAK = 0.986 |  | Reported | Not reported | Total |
| Culture results N (%) | Reported | - | - | - |
|  | Not reported | 1 (0.7%) | 144 (99.3%) | 145 (100.0%) |
|  | Total | 1 (0.69%) | 144 (99.31%) | 145 (100.0%) |
| 1. Peptococcus* |  | Molecular results | |  |
| Kappa: Undefined  PABAK = 0.986 |  | Reported | Not reported | Total |
| Culture results N (%) | Reported | - | 1 (0.7%) | 1 (0.69%) |
|  | Not reported | - | 144 (99.3%) | 144 (99.31%) |
|  | Total | . (.%) | 145 (100.0%) | 145 (100.0%) |
| 1. Dialister* |  | Molecular results | |  |
| Kappa: Undefined  PABAK = 0.986 |  | Reported | Not reported | Total |
| Culture results N (%) | Reported | - | - | - |
|  | Not reported | 1 (0.7%) | 144 (99.3%) | 145 (100.0%) |
|  | Total | 1 (0.69%) | 144 (99.31%) | 145 (100.0%) |
| 1. Aggregatibacter |  | Molecular results | |  |
| Kappa: Undefined  PABAK = 0.986 |  | Reported | Not reported | Total |
| Culture results N (%) | Reported | 1 (0.7%) | 144 (99.3%) | 145 (100.0%) |
|  | Not reported | 1 (0.69%) | 144 (99.31%) | 145 (100.0%) |
|  | Total | 1 (0.7%) | 144 (99.3%) | 145 (100.0%) |
| 1. Moraxella |  | Molecular results | |  |
| Kappa: Undefined  PABAK = 0.986 |  | Reported | Not reported | Total |
| Culture results N (%) | Reported | - | - | - |
|  | Not reported | 1 (0.7%) | 144 (99.3%) | 145 (100.0%) |
|  | Total | 1 (0.69%) | 144 (99.31%) | 145 (100.0%) |

| Genus | Reported present by either method | Reported Present by molecular method | Reported Present by C&S method | Difference in reported presence,(95%CI)(Molecular–C&S) | Agreement (%,95%CI) | Disagreement (%,95%CI) | Cohen's kappa (95%CI) | PABAK |
| --- | --- | --- | --- | --- | --- | --- | --- | --- |
| One or more genus reported | 145 (100.0%) | 145 (100.0%) | 108 (74.5%) | 25.5% (18.0% to 32.3%) | 74.5% (66.6 to 81.4) | 25.5% (18.6 to 33.4) | - | 0.409 |
| Staphylococcus | 102 (70.3%) | 79 (54.5%) | 79 (54.5%) | 0.0% (-7.6% to 7.6%) | 68.3% (60.0 to 75.7) | 31.7% (24.3 to 40.0) | 0.36 (0.21 to 0.51) | 0.366 |
| Corynebacterium | 54 (37.2%) | 47 (32.4%) | 15 (10.3%) | 22.1% (14.2% to 29.3%) | 68.3% (60.0 to 75.7) | 31.7% (24.3 to 40.0) | 0.12 (-0.02 to 0.26) | 0.366 |
| Streptococcus | 54 (37.2%) | 48 (33.1%) | 31 (21.4%) | 11.7% (5.0% to 18.2%) | 80.0% (72.6 to 86.2) | 20.0% (13.8 to 27.4) | 0.50 (0.35 to 0.66) | 0.600 |
| Anaerococcus* | 39 (26.9%) | 38 (26.2%) | 1 (0.7%) | 25.5% (17.9% to 32.4%) | 73.1% (65.1 to 80.1) | 26.9% (19.9 to 34.9) | -0.01 (-0.04 to 0.01) | 0.462 |
| Finegoldia* | 34 (23.4%) | 33 (22.8%) | 1 (0.7%) | 22.1% (14.8% to 28.7%) | 76.6% (68.8 to 83.2) | 23.4% (16.8 to 31.2) | -0.01 (-0.04 to 0.01) | 0.531 |
| Peptoniphilus* | 31 (21.4%) | 30 (20.7%) | 1 (0.7%) | 20.0% (13.0% to 26.5%) | 78.6% (71.0 to 85.0) | 21.4% (15.0 to 29.0) | -0.01 (-0.04 to 0.01) | 0.572 |
| Enterobacteriaceae.unclassified | 31 (21.4%) | 13 (9.0%) | 21 (14.5%) | -5.5% (-12.0% to 1.1%) | 80.7% (73.3 to 86.8) | 19.3% (13.2 to 26.7) | 0.07 (-0.11 to 0.26) | 0.614 |
| Escherichia.Shigella | 20 (13.8%) | 13 (9.0%) | 12 (8.3%) | 0.7% (-4.5% to 5.9%) | 89.7% (83.5 to 94.1) | 10.3% (5.9 to 16.5) | 0.34 (0.09 to 0.60) | 0.793 |
| Proteus | 19 (13.1%) | 11 (7.6%) | 14 (9.7%) | -2.1% (-6.9% to 2.9%) | 91.0% (85.2 to 95.1) | 9.0% (4.9 to 14.8) | 0.43 (0.18 to 0.69) | 0.821 |
| Prevotella* | 15 (10.3%) | 15 (10.3%) | - | 10.3% (5.0% to 15.4%) | 89.7% (83.5 to 94.1) | 10.3% (5.9 to 16.5) | - | 0.793 |
| Enterococcus | 15 (10.3%) | 9 (6.2%) | 9 (6.2%) | 0.0% (-4.7% to 4.7%) | 91.7% (86.0 to 95.7) | 8.3% (4.3 to 14.0) | 0.29 (-0.00 to 0.58) | 0.834 |
| Pseudomonas | 14 (9.7%) | 12 (8.3%) | 10 (6.9%) | 1.4% (-2.3% to 5.0%) | 95.9% (91.2 to 98.5) | 4.1% (1.5 to 8.8) | 0.71 (0.48 to 0.93) | 0.917 |
| Porphyromonas* | 12 (8.3%) | 12 (8.3%) | - | 8.3% (3.4% to 12.9%) | 91.7% (86.0 to 95.7) | 8.3% (4.3 to 14.0) | - | 0.834 |
| Klebsiella | 12 (8.3%) | - | 12 (8.3%) | -8.3% (-12.9% to -3.4%) | 91.7% (86.0 to 95.7) | 8.3% (4.3 to 14.0) | - | 0.834 |
| Helcococcus* | 11 (7.6%) | 10 (6.9%) | 1 (0.7%) | 6.2% (1.5% to 10.7%) | 92.4% (86.8 to 96.2) | 7.6% (3.8 to 13.2) | -0.01 (-0.04 to 0.01) | 0.848 |
| Morganella | 11 (7.6%) | 5 (3.4%) | 7 (4.8%) | -1.4% (-5.8% to 3.1%) | 93.1% (87.7 to 96.6) | 6.9% (3.4 to 12.3) | 0.13 (-0.16 to 0.42) | 0.862 |
| Enterobacter | 10 (6.9%) | - | 10 (6.9%) | -6.9% (-11.2% to -2.4%) | 93.1% (87.7 to 96.6) | 6.9% (3.4 to 12.3) | - | 0.862 |
| Alcaligenes | 9 (6.2%) | 9 (6.2%) | - | 6.2% (1.9% to 10.4%) | 93.8% (88.5 to 97.1) | 6.2% (2.9 to 11.5) | - | 0.876 |
| Providencia | 9 (6.2%) | 2 (1.4%) | 7 (4.8%) | -3.4% (-7.7% to 0.9%) | 93.8% (88.5 to 97.1) | 6.2% (2.9 to 11.5) | -0.02 (-0.05 to 0.00) | 0.876 |
| Serratia | 9 (6.2%) | 1 (0.7%) | 8 (5.5%) | -4.8% (-9.0% to -0.5%) | 93.8% (88.5 to 97.1) | 6.2% (2.9 to 11.5) | -0.01 (-0.03 to 0.01) | 0.876 |
| Haemophilus | 9 (6.2%) | 9 (6.2%) | - | 6.2% (1.9% to 10.4%) | 93.8% (88.5 to 97.1) | 6.2% (2.9 to 11.5) | - | 0.876 |
| Peptostreptococcus* | 8 (5.5%) | 7 (4.8%) | 1 (0.7%) | 4.1% (0.0% to 8.2%) | 94.5% (89.4 to 97.6) | 5.5% (2.4 to 10.6) | -0.01 (-0.03 to 0.01) | 0.890 |
| Cutibacterium* | 7 (4.8%) | 7 (4.8%) | - | 4.8% (0.9% to 8.6%) | 95.2% (90.3 to 98.0) | 4.8% (2.0 to 9.7) | - | 0.903 |
| Veillonella* | 6 (4.1%) | 6 (4.1%) | - | 4.1% (0.4% to 7.7%) | 95.9% (91.2 to 98.5) | 4.1% (1.5 to 8.8) | - | 0.917 |
| Fusobacterium* | 6 (4.1%) | 6 (4.1%) | - | 4.1% (0.4% to 7.7%) | 95.9% (91.2 to 98.5) | 4.1% (1.5 to 8.8) | - | 0.917 |
| Acinetobacter | 6 (4.1%) | 5 (3.4%) | 1 (0.7%) | 2.8% (-0.9% to 6.4%) | 95.9% (91.2 to 98.5) | 4.1% (1.5 to 8.8) | -0.01 (-0.03 to 0.01) | 0.917 |
| Actinomyces | 5 (3.4%) | 3 (2.1%) | 2 (1.4%) | 0.7% (-2.8% to 4.1%) | 96.6% (92.1 to 98.9) | 3.4% (1.1 to 7.9) | -0.02 (-0.03 to -0.00) | 0.931 |
| Bacteroides* | 4 (2.8%) | 4 (2.8%) | - | 2.8% (-0.5% to 5.9%) | 97.2% (93.1 to 99.2) | 2.8% (0.8 to 6.9) | - | 0.945 |
| Brevibacterium | 3 (2.1%) | 3 (2.1%) | - | 2.1% (-0.9% to 5.0%) | 97.9% (94.1 to 99.6) | 2.1% (0.4 to 5.9) | - | 0.959 |
| Micrococcaceae.unclassified | 3 (2.1%) | 2 (1.4%) | 1 (0.7%) | 0.7% (-2.3% to 3.6%) | 97.9% (94.1 to 99.6) | 2.1% (0.4 to 5.9) | -0.01 (-0.02 to 0.00) | 0.959 |
| Parvimonas* | 3 (2.1%) | 2 (1.4%) | 1 (0.7%) | 0.7% (-2.3% to 3.6%) | 97.9% (94.1 to 99.6) | 2.1% (0.4 to 5.9) | -0.01 (-0.02 to 0.00) | 0.959 |
| Micrococcus | 2 (1.4%) | 1 (0.7%) | 1 (0.7%) | 0.0% (-2.6% to 2.6%) | 98.6% (95.1 to 99.8) | 1.4% (0.2 to 4.9) | -0.01 (-0.02 to 0.00) | 0.972 |
| Campylobacter | 2 (1.4%) | 2 (1.4%) | - | 1.4% (-1.3% to 4.0%) | 98.6% (95.1 to 99.8) | 1.4% (0.2 to 4.9) | - | 0.972 |
| Gemella | 2 (1.4%) | 2 (1.4%) | - | 1.4% (-1.3% to 4.0%) | 98.6% (95.1 to 99.8) | 1.4% (0.2 to 4.9) | - | 0.972 |
| Ezakiella* | 2 (1.4%) | 1 (0.7%) | 1 (0.7%) | 0.0% (-2.6% to 2.6%) | 98.6% (95.1 to 99.8) | 1.4% (0.2 to 4.9) | -0.01 (-0.02 to 0.00) | 0.972 |
| Pasteurella | 2 (1.4%) | 2 (1.4%) | - | 1.4% (-1.3% to 4.0%) | 98.6% (95.1 to 99.8) | 1.4% (0.2 to 4.9) | - | 0.972 |
| Actinotignum | 1 (0.7%) | 1 (0.7%) | - | 0.7% (-1.6% to 3.0%) | 99.3% (96.2 to 100.0) | 0.7% (0.0 to 3.8) | - | 0.986 |
| Arcanobacterium | 1 (0.7%) | 1 (0.7%) | - | 0.7% (-1.6% to 3.0%) | 99.3% (96.2 to 100.0) | 0.7% (0.0 to 3.8) | - | 0.986 |
| Fastidiosipila* | 1 (0.7%) | 1 (0.7%) | - | 0.7% (-1.6% to 3.0%) | 99.3% (96.2 to 100.0) | 0.7% (0.0 to 3.8) | - | 0.986 |
| Peptococcus* | 1 (0.7%) | - | 1 (0.7%) | -0.7% (-3.0% to 1.6%) | 99.3% (96.2 to 100.0) | 0.7% (0.0 to 3.8) | - | 0.986 |
| Dialister* | 1 (0.7%) | 1 (0.7%) | - | 0.7% (-1.6% to 3.0%) | 99.3% (96.2 to 100.0) | 0.7% (0.0 to 3.8) | - | 0.986 |
| Aggregatibacter | 1 (0.7%) | 1 (0.7%) | - | 0.7% (-1.6% to 3.0%) | 99.3% (96.2 to 100.0) | 0.7% (0.0 to 3.8) | - | 0.986 |
| Moraxella | 1 (0.7%) | 1 (0.7%) | - | 0.7% (-1.6% to 3.0%) | 99.3% (96.2 to 100.0) | 0.7% (0.0 to 3.8) | - | 0.986 |
| Brachybacterium | - | - | - | 0.0% (-1.9% to 1.9%) | 100.0% (97.5 to 100.0) | 0.0% (0.0 to 2.5) | - | . |
| Dermabacter | - | - | - | 0.0% (-1.9% to 1.9%) | 100.0% (97.5 to 100.0) | 0.0% (0.0 to 2.5) | - | . |
| Kocuria | - | - | - | 0.0% (-1.9% to 1.9%) | 100.0% (97.5 to 100.0) | 0.0% (0.0 to 2.5) | - | . |
| Capnocy to phaga* | - | - | - | 0.0% (-1.9% to 1.9%) | 100.0% (97.5 to 100.0) | 0.0% (0.0 to 2.5) | - | . |
| Sphingobacterium* | - | - | - | 0.0% (-1.9% to 1.9%) | 100.0% (97.5 to 100.0) | 0.0% (0.0 to 2.5) | - | . |
| Granulicatella | - | - | - | 0.0% (-1.9% to 1.9%) | 100.0% (97.5 to 100.0) | 0.0% (0.0 to 2.5) | - | . |
| Clostridium.sensu.stric to .1* | - | - | - | 0.0% (-1.9% to 1.9%) | 100.0% (97.5 to 100.0) | 0.0% (0.0 to 2.5) | - | . |
| Neisseria | - | - | - | 0.0% (-1.9% to 1.9%) | 100.0% (97.5 to 100.0) | 0.0% (0.0 to 2.5) | - | . |
| Stenotrophomonas | - | - | - | 0.0% (-1.9% to 1.9%) | 100.0% (97.5 to 100.0) | 0.0% (0.0 to 2.5) | - | . |

Note: Genera not detected by either method in any of the 145 participants are listed at the end of the table. These genera were reported as 'not present' by both C&S and molecular methods for all participants.

References

1. Sloan TJ, Turton JC, Tyson J, et al. Examining diabetic heel ulcers through an ecological lens: microbial community dynamics associated with healing and infection. *J Med Microbiol*. Feb 2019;68(2):230-240. doi:10.1099/jmm.0.000907

2. Robeson MS, 2nd, O'Rourke DR, Kaehler BD, et al. RESCRIPt: Reproducible sequence taxonomy reference database management. *PLoS Comput Biol*. Nov 2021;17(11):e1009581. doi:10.1371/journal.pcbi.1009581

3. Bokulich NA, Kaehler BD, Rideout JR, et al. Optimizing taxonomic classification of marker-gene amplicon sequences with QIIME 2’s q2-feature-classifier plugin. *Microbiome*. 2018/05/17 2018;6(1):90. doi:10.1186/s40168-018-0470-z
